# Multi-omic serum biomarkers for prognosis of disease progression in prostate cancer

**DOI:** 10.1186/s12967-019-02185-y

**Published:** 2020-01-07

**Authors:** Michael A. Kiebish, Jennifer Cullen, Prachi Mishra, Amina Ali, Eric Milliman, Leonardo O. Rodrigues, Emily Y. Chen, Vladimir Tolstikov, Lixia Zhang, Kiki Panagopoulos, Punit Shah, Yongmei Chen, Gyorgy Petrovics, Inger L. Rosner, Isabell A. Sesterhenn, David G. McLeod, Elder Granger, Rangaprasad Sarangarajan, Viatcheslav Akmaev, Alagarsamy Srinivasan, Shiv Srivastava, Niven R. Narain, Albert Dobi

**Affiliations:** 1 BERG LLC, Framingham, MA USA; 2 Henry Jackson Foundation for the Advancement of Military Medicine, Bethesda, MD USA; 3 Center for Prostate Disease Research, Department of Surgery, Uniformed Services University and the Walter Reed National Military Medical Center, Bethesda, MD USA; 4 Joint Pathology Center, Silver Spring, MD USA

**Keywords:** Bayesian networks, Biochemical recurrence, Biomarkers, Metabolomics, Prostate cancer

## Abstract

**Background:**

Predicting the clinical course of prostate cancer is challenging due to the wide biological spectrum of the disease. The objective of our study was to identify prostate cancer prognostic markers in patients ‘sera using a multi-omics discovery platform.

**Methods:**

Pre-surgical serum samples collected from a longitudinal, racially diverse, prostate cancer patient cohort (N = 382) were examined. Linear Regression and Bayesian computational approaches integrated with multi-omics, were used to select markers to predict biochemical recurrence (BCR). BCR-free survival was modeled using unadjusted Kaplan–Meier estimation curves and multivariable Cox proportional hazards analysis, adjusted for key pathologic variables. Receiver operating characteristic (ROC) curve statistics were used to examine the predictive value of markers in discriminating BCR events from non-events. The findings were further validated by creating a training set (N = 267) and testing set (N = 115) from the cohort.

**Results:**

Among 382 patients, 72 (19%) experienced a BCR event in a median follow-up time of 6.9 years. Two proteins—Tenascin C (TNC) and Apolipoprotein A1V (Apo-AIV), one metabolite—1-Methyladenosine (1-MA) and one phospholipid molecular species phosphatidic acid (PA) 18:0-22:0 showed a cumulative predictive performance of AUC = 0.78 [OR (95% CI) = 6.56 (2.98–14.40), *P* < 0.05], in differentiating patients with and without BCR event. In the validation set all four metabolites consistently reproduced an equivalent performance with high negative predictive value (NPV; > 80%) for BCR. The combination of pTstage and Gleason score with the analytes, further increased the sensitivity [AUC = 0.89, 95% (CI) = 4.45–32.05, *P* < 0.05], with an increased NPV (0.96) and OR (12.4) for BCR. The panel of markers combined with the pathological parameters demonstrated a more accurate prediction of BCR than the pathological parameters alone in prostate cancer.

**Conclusions:**

In this study, a panel of serum analytes were identified that complemented pathologic patient features in predicting prostate cancer progression. This panel offers a new opportunity to complement current prognostic markers and to monitor the potential impact of primary treatment versus surveillance on patient oncological outcome.

## Background

Prostate cancer is the second leading cause of death in men with cancer in the United States [1]. Approximately 30–40% of patients treated with radical prostatectomy (RP) for clinically localized prostate cancer will experience disease progression indicated by rising post-surgery serum prostate specific antigen (PSA) levels. Therefore, discovery of early biomarkers for prostate cancer progression are crucial to predict the risk of relapse and to temper active monitoring using PSA.

The use of early prognostic markers of the disease remains a challenge. Commonly used prognostic markers such as diagnostic PSA, biopsy grade and clinical stage have limited value in predicting which patients will develop metastatic prostate cancer. Intensive efforts have led to the development of new biomarkers for early detection and prognosis of prostate cancer. These biomarkers include pre-diagnostic urine-based assays (PCA3, T2-ERG, Exosome DX, Select MDx and Prostarix), serum-based assays for PSA derivatives, and diagnostic biopsy tissue-based assays (Oncotype DX, Prolaris, Decipher and ProMark assays) [2–10]. While most of the pre-treatment assays rely on biopsy tissue, the rise in post-treatment serum PSA and detection of metastasis using imaging modalities remains the “gold standard” for monitoring disease progression.

Liquid biopsies are among the most preferred tests as they are non-invasive and rapidly performed, in comparison to tissue biopsies. However, there are limitations in the diagnostic and prognostic performance of blood-based biomarkers of prostate cancer. Current methods include PSA in combination with digital rectal exam (DRE) [11], the Prostate Health Index (PHI) which analyzes a combination of free-PSA (fPSA), total PSA (tPSA), and [-2]proPSA to predict risk of Gleason ≥ 7 disease on biopsy [12]. The 4 K score (a combination of four kallikrein proteins, tPSA, fPSA, intact PSA and hK2) [13], and CellSearch™ CTC was designed to measure circulating tumor cells (CTCs) for monitoring treatment response in advanced disease [14]. Of interest, recent studies conducted on surgically resectable cancers (ovary, liver, stomach, pancreas, esophagus, colorectum, lung and breast) established a multi-analyte blood test (CancerSEEK) for assessment of the levels of circulating proteins and mutated cell-free DNAs, to detect the type of cancer and their metastatic localization [15, 16]. While most of the liquid biopsy prognostic panels developed to date are using one type of analyte, there are very few which are based on multiple analytes [15, 16], especially for prostate cancer.

In the current study, we sought to identify serum biomarkers to complement pathological parameters in predicting disease progression. This was addressed by using sera donated by a racially diverse cohort of military health care beneficiaries in an equal access health care system, followed over 20 years.

## Methods

### Study design and participants

**Fig. 1:**
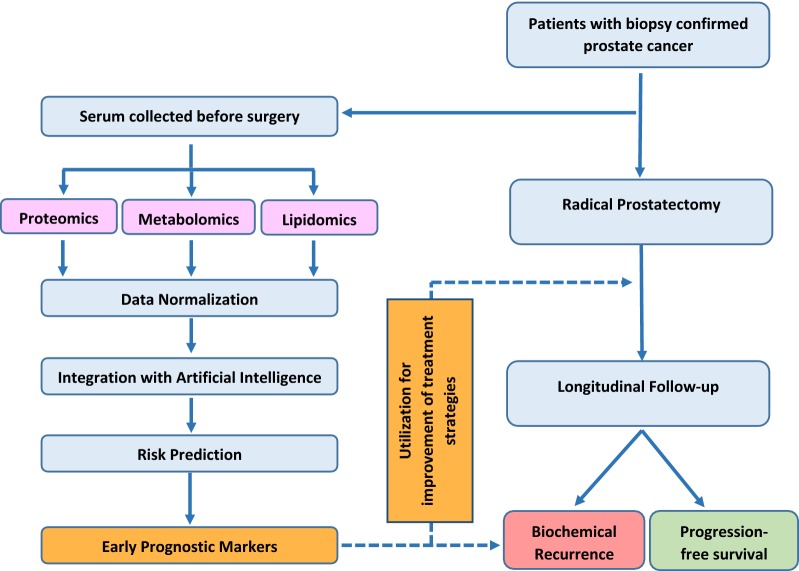
Conceptual schematic describes approach for integrating multi-omics and artificial intelligence for biomarker discovery from presurgical serum for prognosis of prostate cancer progression

In this retrospective cohort study, patients enrolled in both the Center for Prostate Disease Research (CPDR) biospecimen databank and the multi-center national clinical database, were eligible. Both databases have been approved by the institutional review boards (IRB) at the Walter Reed National Military Medical Center (WRNMMC) and the Uniformed Services University of the Health Sciences (USUHS) in Bethesda, Maryland, respectively. Eligibility was further restricted to subjects who underwent RP without neoadjuvant therapy for treatment of biopsy-confirmed prostate cancer between 1997 through 2014 and donated a serum sample at time of RP. Detailed information on patient demographic, clinical, pathologic, treatment, and cancer outcomes was obtained as part of routine data abstraction activities for the CPDR multi-center national database (Additional file 1: Table S1). Patient’s characteristics of interest included: age at RP (years), self-reported race (African American, AA; Caucasian American, CA), PSA level (ng/mL) at time of prostate cancer diagnosis, pathologic T stage (pT2, pT3–4), pathologic Gleason sum (≤ 6; 3 + 4; 4 + 3 and 8 − 10), NCCN Risk stratum and surgical margin status (positive, negative). Patient follow up time was calculated as days elapsed between RP date and the last known medical visit. The concept used for the serum biomarker discovery, using the OMICS platform for prognosis of prostate cancer progression was as shown in Fig. 1.

### Primary study outcome

The primary study endpoint was biochemical recurrence (BCR). A BCR event was defined in the following manner: a post-operative PSA level ≥ 0.2 ng/mL followed by a successive, confirmatory PSA level ≥ 0.2 or the initiation of salvage radiation or hormonal therapy after a rising PSA level ≥ 0.1. Any PSA value taken within 8 weeks after RP were not considered due to known PSA fluctuations proximate to the RP date. Patients who had salvage therapy *without* a rising PSA ≥ 0.1 were classified as a non-BCR event and censored at the date of the initiation of salvage therapy. Patients who had an initial PSA ≥ 0.2 ng/mL but no confirmatory PSA ≥ 0.2 and no initiation of salvage therapy, were classified as a non-BCR event and censored at the last known date of PSA of < 0.2 ng/ml. Distant metastasis (DM) was assessed by a systematic review of the electronic medical record and physician-ordered scans appropriate for confirming metastasis. Patients who are lost to follow up or who died without any evidence of distant metastasis are censored as non-events on the date of last known medical visit or date of death, respectively. The quality of serum samples was examined before their analysis in multi-omics platforms. No hemolysis or lipemia was observed in any of the analyzed samples.

### Metabolomics, proteomics and lipidomics analysis

Blood (12 ml) was drawn into two serum separation tiger-topped tubes (2 × 6 ml) and allowed to clot for 30 min at room temperature and centrifuged for 20 min at 1617*g*. Serum were aliquoted and stored at − 80 °C for Omics analysis. A one ml aliquot was thawed and was simultaneously analyzed for proteins, structural and signaling lipids, and metabolites, on their respective analysis platforms using global proteomics, MS/MS^ALL^ shotgun lipidomic, high resolution targeted MS/MS signaling lipidomics, targeted hydrophilic interactive liquid chromatography mass spectrometry (HIILC–MS/MS), reverse phase high resolution liquid chromatography mass spectrometry (LC-MS), and volatile metabolite analysis using gas chromatography- time of flight- mass spectrometry (GC-TOF–MS) platforms. Detailed analysis is provided in Additional file 1: Materials and Methods. Data from individual platforms were streamed into Interrogative Biology platform, Bayesian Network Inference (BNI) modules and statistical/regression models to derive the prognostic risk of BCR or DM.

### Feature selection

Raw data from 2205 analytes (lipids, metabolites and proteins) were pre-processed before analysis. Briefly, each analyte was standardized and 261 analytes (11.8%) were removed for containing more than 50% of missing data. When applicable, the missing data was imputed by random values around the lowest detection range within each Omic technology. Univariable screening of the analytes’ BCR/non-BCR fold change was conducted on the non-imputed data using non-parametric permutation testing [17]. False discovery rate (FDR) [18] was applied to adjust the analytes’ P-values with a cutoff at 0.05. The search space was further expanded by a multivariable analysis of the top 55 ranked analytes using elastic-net coupled with bootstrapping [19, 20]. Given the solution paths from bootstrapping 200 times, the analytes were ranked by the averaged selection rate at each given size of selected variables, where a single selection rate was calculated as the percentage of selection among the number of bootstrapped sampling. Therefore, the best analyte had the highest selection rate when one analyte was selected as a biomarker, and the second analyte had the highest averaged selection rate among the remaining analytes given a size of two, and so forth, to all the analytes processed in the multivariable analysis. The selection of clinical variables was based on causal graphs (networks) generated by BERG’s AI platform bAIcis^®^ which relies on Bayesian network methods to learn which directed acyclic graph is most likely given the provided data [21]. In order to identify potential causal drivers of the BCR status, an ensemble model from all variables was generated using bAIcis^®^, and the clinical variables directly connected to the outcome of interest were selected for further exploration. Based on the analytes rank and the selection rate at each size, hierarchical clustering grouped analytes with similar selection performance, and top-ranked clusters were considered for further analysis.

Receiver operating characteristic (ROC) curve analysis was performed to access the performance of each biomarker based on the logistic regression models (BCR versus non-BCR). Area under the curve (AUC) statistics were used to assess the predictive value of the selected analytes. Further details provided in Additional file 1: Materials and Methods.

### Development of a biomarker classifier and optimal cutoff of each marker in predicting disease progression in training and testing sets

This 382 patient cohort was randomly split into training and testing cohorts (i.e., 70% vs. 30% of study sample) following the methods as described in Gholami et al. [22]. The training cohort consisted of 267 patients (50 BCR, 217 non-BCR events), while the testing cohort consisted of 115 patients (22 BCR, 93 non-BCR). Multivariable logistic regression model was used to determine the parameter estimate of each marker in the training cohort; those estimates were then applied to construct a 4-marker panel classifier in the testing cohort. The prediction accuracy of the 4-marker classifier in predicting disease progression (combined with pathological variables) was examined using ROC analysis. Bootstrapped, univariable logistic regression model (with 1000 replicates) was used to search for the optimal cutoff of each marker in predicting BCR first using the training cohort, then in the testing cohort. The optimal threshold was defined as a cut point which maximized sensitivity, achieving an NPV > 80% and a specificity (SPC) > 35%.

### Survival analysis

Kaplan–Meier (KM) analysis and log-rank test were used to identify the 4 markers in predicting BCR-free or DM-free survival. The markers were also evaluated by adding to standard of care (SOC) variables (pathological T stage, Gleason sum and surgical margin). Multivariable-Cox proportional hazard analysis was then used to examine these markers in combination of SOC (BCR-free and DM-free). The proportional hazard assumption of each covariate was checked and met.

## Results

### Study design

The study cohort included a total of 382 patients. Clinicopathological parameters were stratified across the event status: BCR (N = 72) and non-BCR (N = 310) patients. Among the BCR patients, 11 developed distant metastases. The patients included 314 self-reported CA (BCR 17.8%, Non BCR 82.2%) and 68 self-reported AA (BCR 13.5%, Non BCR 76.5%). The median age of the overall cohort at RP was 58.2 years and the median follow-up time was 6.9 years. Other variables were Biopsy Gleason Sum and Pathologic Stage (pT2 and pT3–4), as described in Table 1.

**Table 1:** Descriptive characteristics of the study cohort stratified by event status (N = 382)

Variable	Overall	Non-BCR	BCR^a^
N (%)	382	310 (81.2)	72 (18.8)
Age at RP (years)			
Mean (SD)	58.2 (8.3)	57.9 (8.3)	59.4 (8.3)
Race—N (%)			
CA & Other	314 (82.2)	258 (82.2)	56 (17.8)
AA	68 (17.8)	52 (76.5)	16 (13.5)
Pathological T stage—N (%)			
pT2	259 (72.8)	232 (89.6)	27 (10.4)
pT3–4	97 (27.2)	62 (63.9)	35 (36.1)
Gleason sum—N (%)			
3 + 3	196 (54.3)	178 (90.8)	18 (9.2)
3 + 4	105 (29.1)	91 (86.7)	14 (13.3)
4 + 3/8 − 10	60 (16.6)	31 (51.7)	29 (48.3)
Surgical margin—N (%)			
Negative	311 (81.8)	263 (84.5)	48 (15.4)
Positive	69 (18.2)	46 (66.7)	23 (33.3)
Follow up time (years)			
Median (range)	6.9 (0.2–18.6)	6.6 (0.2–18.6)	8.2 (1.5–17.8)

^a^Among 72 patients with BCR, 11 developed distant metastasis after BCR

### Identification of BCR-status predictors

**Fig. 2:**
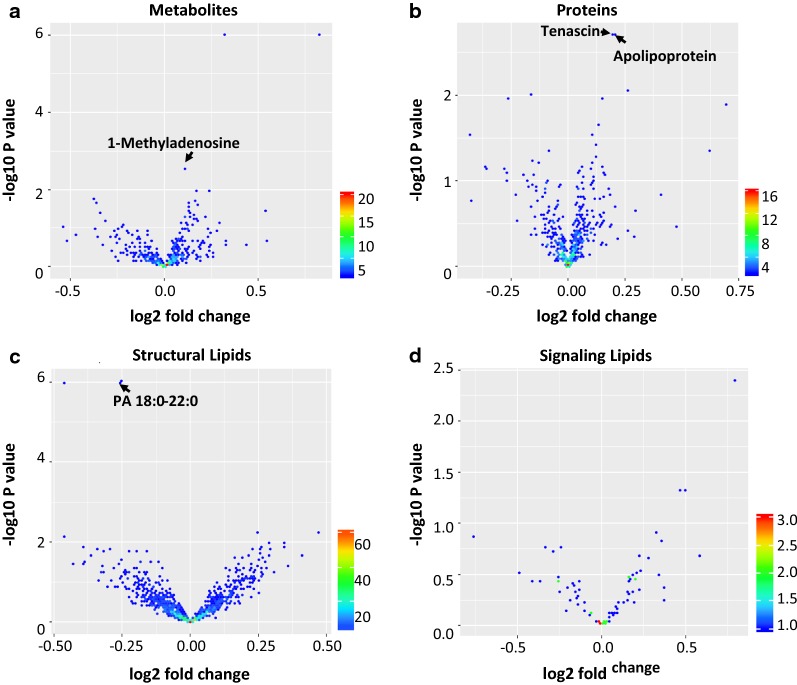
Volcano plots depict the top analytes from serum samples of prostate cancer patients with BCR, run through different mass spectrometry platforms for abundance of **a** metabolites, **b** proteins, **c** structural lipids and **d** signaling lipids. Each dot in the plot represents an analyte. The X-axis represents the fold change of the analytes (log2 scale); the Y-axis representing the P-values in − log10 scale. The color bar represents the number of analytes that had a fold-change and *P*-value in that range. For metabolites: 17 had an unadjusted *P* ≤ 0.05. For proteins: 13 had an unadjusted *P* ≤ 0.05. For structural lipids: 56 had an unadjusted *P* ≤ 0.05. For signaling lipids: 2 had an unadjusted *P* ≤ 0.05

**Fig. 3:**
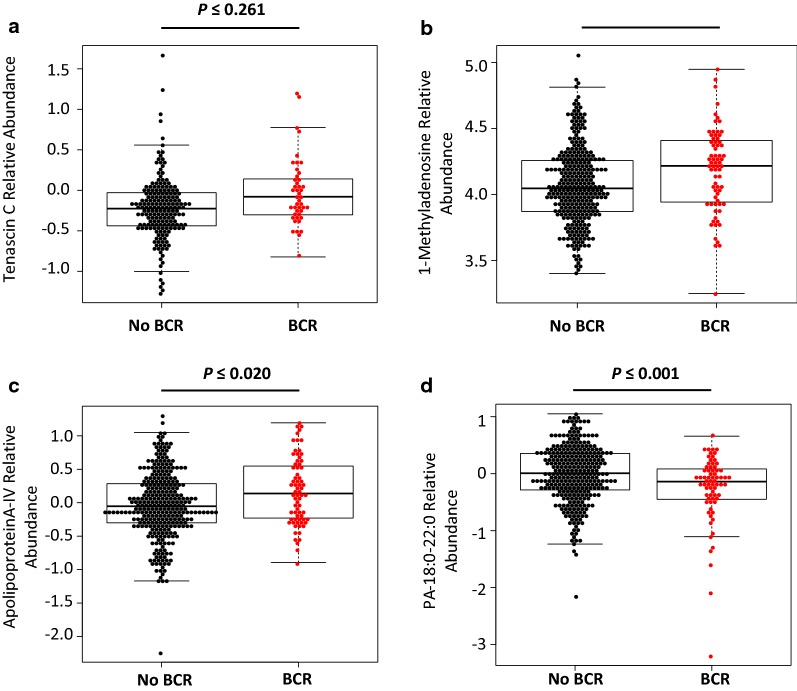
Plots represent specific metabolites and proteins individually with a significant differential abundance selected to form a combinatorial panel for diagnosis or prognosis of prostate cancer. **a** Tenascin C, **b** Apolipoprotein A-IV, **c** 1-methyladenosine and **d** PA-18:0-22:0 were the four chosen analytes. The normalized abundance of the analytes is represented in boxplots. Each dot represents a patient measurement for the stated analyte

Global analyses of the serum proteome, structural lipidome, signal lipidome and metabolome, identified 2205 analytes with differential abundance in BCR vs non-BCR events, as represented through volcano plots (Fig. 2a–d), and were assessed for predictive utility by evaluating a combination of those analytes which complemented each other for prediction of disease progression. Elastic net analysis identified four analytes based on their ability to discriminate between patients with BCR versus non-BCR, two proteins Tenascin C (TNC) and Apolipoprotein A-IV (ApoA-IV), one metabolite, 1-methyladenosine (1-MA), and one lipid molecular species, Phosphatidic Acid (PA) 18:0-22:0, as the best performing analytes that step-wise disqualified analytes which do not provide any additional discriminatory power for prediction of event status (Fig. 3a–d). Data for each of the analytes was recorded as log transformed measurements across OMIC datasets for comparison. The selection of clinical features that could potentially differentiate BCR vs non-BCR status was performed through bAIcis^®^. Based on the topological analysis of the causal network generated by bAIcis^®^, Gleason score and T-stage were inferred as features which drive the BCR status and, hence, were selected for further analysis..

### Performance of the analytes with and without clinical parameters

**Fig. 4:**
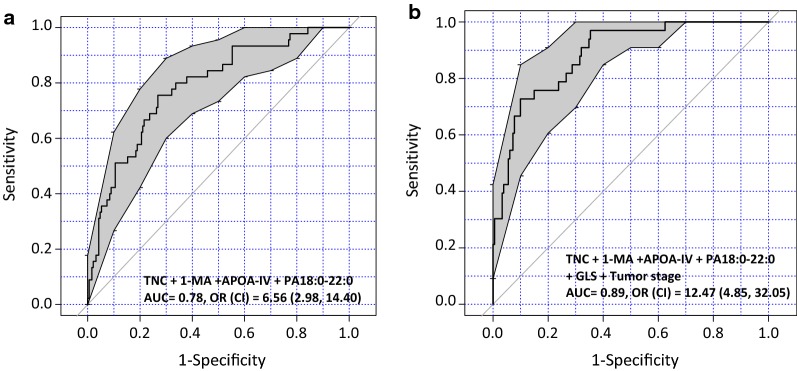
ROC curve analysis of the marker panel alone compared to marker panel plus “standard of care” pathology variables. **a** The analysis demonstrates cumulative sensitivity and specificity of four markers with an AUC = 0.78, OR (CI) = 6.56 (2.98, 14.40), selected as ideal biomarkers for a prognostic test. **b** ROC analysis shows the combined sensitivity and specificity of four markers along with the pathological/clinical features increasing the AUC = 0.89 and OR (CI) = 12.47 (4.85, 32.05) representing an enhanced prognostic test. *1-MA* 1-methyladenosine, *APOA-IV* apolipoprotein A-IV, *AUC* area under curve, *GLS* gleason score, *OR* odds ratio, *TNC* Tenascin C

Analysis of the four-analytes panel yielded an AUC of 0.78 and an OR of 6.56 (*P* ≤ 0.001) in differentiating patients with BCR event from non-events (Fig. 4a). Pathological T stage alone provided an AUC of 0.67 and an OR of 0.21 (*P *= 0.43) and Gleason score yielded an AUC of 0.69 and OR of 2.68 (*P *= 0.27), neither were statistically significant (data not shown). However, combining these two pathological variables with the analyte panel resulted in a AUC of 0.89, PPV of 0.3, NPV of 0.96, and an OR of 12.47 (*P *≤ 0.001), thus demonstrating a robust performance of the panel in combination with clinical pathological features (Fig. 4b).

### Validating the performance of the analytes in the training and test cohort

The findings were validated by creating a training set with BCR (N = 50) vs Non-BCR (N = 217) and testing set BCR (N = 22) vs Non-BCR (N = 93) within the cohort to maintain proportional distribution in the event status (Additional file 1: Table S1) taking into account similar variables (Age at RP, Race). In both the training and testing set cohort, the Pathological T stage and Gleason score were consistent and significantly different across event status (Table 2). We further validated the performance for each of the individual markers TNC, Apo A-IV, 1-MA and PA18:0-22:0, with cut-points optimized for a highest NPV, with specificity > 30% (Table 3), in both the training and testing sets for prediction of disease progression. The AUC values demonstrated moderate sensitivity with high NPVs, having a similar trend in both the sets. A series of multivariable Cox proportional hazards model were examined to consider varying restriction or the minimum follow-up time for non-events. Models for 1-year, 3-year and 5-year follow-up were compared to the overall model and findings were consistent, therefore, the overall model was presented. The multivariable Cox proportional hazards analysis (proportional hazards assumption, tested and met) for the overall model revealed a significant association between Pathological T stage (HR = 2.81, *P *= 0.001), Pathological Gleason sum (HR = 5.57, *P *= 0.001), Apo A-IV (High vs low) (HR = 2.21, *P* 0.017), 1-MA (high vs low) (HR = 2.11, *P *= 0.017), PA-18:0-22:0 (Low vs high) (HR = 2.49, *P *= 0.006) in predicting the BCR free survival (Table 4). While TNC (HR = 0.99, *P *= 0.966) was not predictive of BCR, it was a robust predictor for the unadjusted metastasis-free survival. Adjusted analysis was not performed for distant metastasis- free survival due to a small number of metastatic events.

**Table 2:** Distributions of clinico-pathological variables between non-progression and progression groups among training and testing cohorts

Variable	Training (N = 267)			Testing (N = 115)		
	Non-BCR	BCR	P value	Non-BCR	BCR	*P* value
N	217	50		93	22	
Age at RP (years)	58.1 (8.3)	59.2 (8.3)		57.3 (8.4)	59.9 (8.6)	
Mean (SD)	58.8 (40.7–76.1)	61.2 (42.6–70.6)	0.271	57.5 (40.6–76.9)	62.1 (41.4–70.7)	0.081
Race—N (%)						
CA & Other	179 (82.5)	38 (17.5)		79 (81.4)	18 (18.6)	
AA	38 (76.0)	12 (24.0)		14 (77.8)	4 (22.2)	
Pathological T stage—N (%)						
pT2	164 (89.6)	19 (10.4)		68 (89.5)	8 (10.5)	
pT3–4	43 (62.3)	26 (37.7)	*< 0.001*	19 (67.9)	9 (32.1)	*0.008*
Gleason sum—N (%)						
3 + 3	24 (90.8)	12 (9.2)		60 (90.9)	6 (9.1)	
3 + 4	65 (89.0)	8 (11.0)		26 (81.2)	6 (18.8)	
4 + 3/8 − 10	24 (53.3)	21 (46.7)	*< 0.001*	7 (46.7)	8 (53.3)	*< 0.001*
Surgical margin—N (%)						
Negative	189 (84.8)	34 (15.2)		74 (84.1)	14 (15.9)	
Positive	27 (64.3)	15 (35.7)	*0.001*	19 (70.4)	8 (29.6)	0.114

The statistically significant *P* values (*P* < 0.05) are given in italics

*RP* radical prostatectomy

**Table 3:** Performance of each marker in predicting disease progression among training and testing cohorts

Marker	Training						Testing			
	AUC	Cut point	NPV	Sens	SPC	PPV	NPV	Sens	SPC	PPV
PA-18:0–22:0	0.64	0.11	0.89	0.80	0.39	0.23	0.91	0.77	0.54	0.28
Apolipoprotein A-IV	0.49	− 0.21	0.85	0.72	0.35	0.20	0.85	0.77	0.30	0.21
Tenascin C	0.51	− 0.17	0.82	0.30	0.41	0.19	0.81	0.50	0.49	0.19
1-Methyladenosine	0.59	3.98	0.87	0.76	0.37	0.22	0.80	0.59	0.38	0.18

*AUC* area under curve, *NPV* negative predictive value, *PPV* positive predictive value, *SPC* specificity

**Table 4:** Multivariable Cox proportional hazard model predicting BCR by adding 4 markers to SOC

Variable	HR	95% CI	*P* value
Pathological T stage (T3 vs T2)	2.81	1.49–5.30	*0.001*
Gleason sum (4 + 3/8 − 10 vs 3 + 3/3 + 4)	5.57	3.25–9.56	*< 0.001*
Surgical margin (Pos vs neg)	1.27	0.62–2.57	0.511
PA-18:0–22:0 (Low vs high)	2.49	1.30–4.79	*0.006*
Apolipoprotein A-IV (High vs low)	2.21	1.15–4.24	*0.017*
Tenascin C (High vs low)	0.99	0.56–1.73	0.966
1-Methyladenosine (high vs low)	2.11	1.14–3.92	*0.017*

The statistically significant *P* values (*P* < 0.05) are given in italics

*SOC* standard of care, *HR* hazards ratio, *CI* confidence interval

KM survival curve analysis for the dichotomized marker groups for BCR-free survival demonstrated association between each marker independently, and poor survival in patients with low serum PA-18:0-22:0 (*P *= 0.0005) (Additional file 1: Fig. S1). In KM analysis for metastasis-free survival both TNC (*P *= 0.0227) and PA-18:0-22:0 (*P *= 0.0450) were significant predictors for poor outcome (Additional file 1: Fig. S2). Interestingly, while higher levels of serum TNC, 1-MA and APO-AIV signified the directionality for increased probability of the disease progression, lower levels of PA-18:0-22:0 complemented for an increased probability of the disease progression. Taken together, the 4 analytes in combination with the two pathological parameters (Pathological T stage and Gleason score), robustly enhanced the sensitivity of the panel to detect the BCR in our prostate cancer cohort.

## Discussion

To date, there have been numerous biomarker panels identified for early prognosis of prostate cancer. However, all had some limitations to reach extensive use in clinical practices. The serum PSA test remains as the gold standard for monitoring BCR in patients with primary treatment, suggesting a critical unmet need of biomarkers for early risk stratification in patients with RP. In this regard, a blood-based assay that is more accurate, economical, and can be performed through standard clinical procedures and could be a major advantage in clinical management.

The primary aim of this study was to identify analytes in pre-surgical sera which can serve as predictors of disease progression in patients with prostate cancer, with adjustment for known pathological factors. Towards this goal, a panel of four serum analytes was identified, which in combination with clinical features could serve as early indicators, thus differentiating patients with high risk of BCR from those without risk. We highlight here a combination of four analytes (TNC, ApoA-IV, 1-MA or PA18:0-22:0) with pathologic Gleason score and tumor stage, for prostate cancer prognosis. The strength of our serum multi-analyte panel is the robust performance in predicting BCR-free survival (Negative Predictive Value). A possible reason of the complementary nature of analytes in this panel is the involvement of these analytes in distinct cancer associated pathways. TNC, Apo A-IV and 1-MA have been implicated in aggressive forms of cancers [23–28].

Tenascin C is an extracellular matrix protein with major function in metastasis, initiation [29, 30] and progression [31]. The prognostic value of TNC has been reported for non-small cell lung carcinoma [24], esophageal squamous cell carcinoma [28] and colon cancer [32, 33]. Few studies linked TNC to poor prognosis of prostate cancer [34, 35] which supported the appreciable sensitivity of serum TNC for metastasis, in the present study. Association of apolipoproteins with disease progression was less understood, though few studies demonstrate their association with tumorigenesis and poor prognosis [23, 36, 37]. Apolipoprotein A-IV, was suggested as a marker for ovarian cancer [25, 38] and was highlighted in context of detecting cancer in prostate, lung, pancreatic, uterus and bladder, but had not been assessed for use as a prognostic biomarker [39]. Similarly, 1-methyladenosine is among one of the nucleoside metabolites that was linked to prognosis of breast cancer [40], leukemia and lymphoma [41], genito-urinary cancer, colon, lung and gastric cancers [42] and also an indicator of increased systemic RNA turnover [26]. Thus, the unbiased selection of these analytes in our panel indeed reflects the cancer progression-associated biology in the patient serum. PA18:0–22:0 was found to be an analyte of unknown characteristics, though it complemented the biomarker panel significantly.

The major limitation of this prognostic serum biomarker discovery study is the small number of prostate cancer metastatic events in the cohort. Although BCR may prompt therapeutic intervention, metastasis is a more precisely defined endpoint for prognostication. Another limitation is that serum samples in this study were collected right before radical-prostatectomy. We employed this strategy to increase the odds of prognostic marker discovery. However, assessment of the marker panel in pre- and post-diagnostic settings is warranted. Our findings highlighted multi-analyte discovery in racially diverse populations of AA and CA patients with a healthcare system of equal access. This is the first study from a cohort that had been longitudinally followed up for approximately two decades, and that report a multianalyte biomarkers for prognosis of prostate cancer using multi-OMICS and Bayesian networks. The serum-based four analyte marker and two clinical feature panel defined in this study has promising potential in prognosing progression-free survival and provides new strategy to complement early stages of prostate cancer disease management.

## Conclusion

A serum-based four analyte markers panel (TNC, 1-MA, APOA-IV and PA18:0-22:0) complemented with Gleason score and tumor stage, can be used to predict the recurrence of disease post-surgery and modify treatment strategies to improve the survival of prostate cancer patients.

## Supplementary information

**Additional file 1: Figure S1.** Kaplan–Meier BCR-free survival curves across dichotomized marker levels of (PA)18:0-22:0, Tenascin C, Apolipoprotein A-IV and 1-Methyladenosine. **Figure S2.** Kaplan–Meier metastasis-free survival curves across dichotomized marker levels of (PA)18:0-22:0, Tenascin C, Apolipoprotein A-IV and 1-Methyladenosine. **Table S1.** Clinico-pathological variable distribution between training and testing cohorts.

## Data Availability

All the data generated and analyzed during this study are included in the manuscript and the additional materials.

## Abbreviations

BCR: biochemical recurrence
ROC: receiver operating characteristic
AUC: area under curve
TNC: Tenascin C
APOA-IV: apolipoprotein A-IV
1-MA: 1-methyladenosine
RP: radical prostatectomy
DM: distant metastasis
AA: African American
CA: caucasian
DRE: digital rectal examination
HR: hazards ratio
OR: odds ratio
PPV: positive predictive value
NPV: negative predictive value
PSA: prostate specific antigen

## Disclaimer

The opinions or assertions contained herein are the private ones of the author/speaker and are not to be construed as official or reflecting the views of the Department of Defense, the Uniformed Services University of the Health Sciences or any other agency of the U.S. Government. The identification of specific products, scientific instrumentation, or organization is considered an integral part of the scientific endeavor and does not constitute endorsement or implied endorsement on the part of the author, DoD, or any component agency.
