# Supplementary material for: Multi-omic serum biomarkers for prognosis of disease progression in prostate cancer
Source: J Transl Med. 2020 Jan 7;18:10. doi: 10.1186/s12967-019-02185-y (PMC6945688; doi:10.1186/s12967-019-02185-y)

**Additional Materials**

**Serum Metabolomics Analysis**

Serum aliquots (75 μl) were placed in pre-chilled (-80°C) 1.5 mL Eppendorf tubes. Next, 400 µL of a pre-chilled (-20°C) mixture of acetonitrile, isopropanol, and deionized water in proportion 3:3:2 (v/v/v) was added. Samples were vortexed for 10 sec and left in freezer at -20^o^C overnight. Samples were further centrifuged at 4°C at 12,000 g for 3 minutes. Supernatants were transferred into LC-MS vials or into 0.5 mL Eppendorf tubes. Serum extracts were then divided in to three parts: 75 μL to be further dried and derivatized with MSTFA and methoxyamine for gas chromatography combined with time-of-flight mass spectrometry, using a time-of-flight Pegasus HT mass spectrometer (LECO, St. Josephs, MI, USA), 200 μL for reversed phase liquid chromatography coupled with high-resolution mass spectrometry using a TripleTOF® 6600 (SCIEX, Framingham, MA, USA) (to be further dried and reconstituted with 40 μL mixture of 0.1% formic acid and acetonitrile in proportion 9:1 v.v.), and 100 μL for hydrophilic interaction chromatography with liquid chromatography and tandem mass-spectrometry using a TripleQuad 5500 System (SCIEX, Framingham, MA, USA). A standard quality control sample containing a mixture of amino and organic acids was injected daily to monitor mass spectrometer response. A pooled quality control sample was then obtained by taking an aliquot of the same volume of all samples from the study and injected daily with a batch of analyzed samples and to determine the optimal dilution of the batch samples and to validate metabolite identification and peak integration.

**Structural Lipidomic Profiling**

A cocktail of deuterium-labeled and odd chain phospholipid standards from diverse lipid classes was added to 25 µL of serum. Standards were chosen so that they represented each lipid class and were at designated concentrations were chosen to provide the most accurate quantitation and dynamic range for each lipid species. Next, 4 mL chloroform:methanol (1:1, v/v) was added to each sample and the lipid extraction were performed as previously described [12,13]. Lipid extraction was automated using a customized sequence on a Hamilton Robotics STARlet system (Hamilton, Reno, NV) to meet the high-throughput requirements. Lipid extracts were dried under nitrogen and reconstituted in 68 µl chloroform:methanol (1:1, v/v). Samples were flushed with nitrogen and stored at -20 °C. Samples were diluted 50 times in isopropanol:methanol:acetonitrile:water (3:3:3:1, by volume) with 2 mM ammonium acetate in order to optimize ionization efficiency in positive and negative modes. Electrospray ionization-MS was performed on a TripleTOF® 5600^+^ (SCIEX, Framingham, MA), coupled to a customized direct injection loop on an Ekspert microLC200 system (SCIEX). Next, 50 µL of sample was injected at a flow-rate of 6 µL/min. Lipids were analyzed using a customized data independent analysis strategy on the TripleTOF® 5600^+^ allowing for MS/MS^ALL^ high resolution and high mass accuracy analysis. Quantification was performed using an in-house library on MultiQuant™ software (SCIEX).

**Mediator Lipidomic profiling**

A mixture of deuterium-labeled internal standards was added to aliquots of 100 µL serum, followed by 3x volume of sample of cold methanol (MeOH). Samples were vortexed for 5 min and stored at −20 °C overnight. Cold samples were centrifuged at 14,000g at 4 °C for 10 min, and the supernatant was then transferred to a new tube and 3 mL of acidified H2O (pH 3.5) was added to each sample prior to C18 SPE and performed as previously described (1). The methyl formate fractions were collected, dried under nitrogen, and reconstituted in 50 µL MeOH: H2O (1:1, by vol). Samples were transferred to 0.5 mL tubes and centrifuged at 20,000g at 4°C for 10 min. Thirty-five microliters of supernatant was transferred to LC–MS vials for analysis using the BERG LC–MS/MS mediator lipidomics platform. Separation of signaling lipids was performed on an Ekspert MicroLC 200 system (Eksigent Technologies) with a Synergi™ Fusion-RP capillary C18 column (150 × 0.5 mm, 4 µm; Phenomenex Inc., Torrance, CA, USA) heated to 40°C. A sample volume of 11 µL was injected at a flow rate of 20 µL/min. Lipids were separated using mobile phases A (100 % H2O, 0.1 % acetic acid) and B (100 % MeOH, 0.1 % acetic acid) with a gradient starting at 60% B for 0.5 min, steadily increasing to 80% B by 5 min, reaching 95% B by 9 min, holding for 1 min, and then decreasing to 60% B by 12 min. MS analysis was performed on a SCIEX TripleTOF® 5600+ system using the MRMHR strategy consisting of a TOF MS experiment looped with multiple MS/MS experiments. MS spectra were acquired in high-resolution mode (>30,000) using a 100-ms accumulation time per spectrum. Full-scan MS/MS was acquired in high sensitivity mode, with an accumulation time optimized per cycle. Collision energy was set using rolling collision energy with a spread of 15V. The identity of a component was confirmed using PeakView® software (SCIEX), and quantification was performed using MultiQuant™ software (SCIEX).

**Serum Proteomic Analysis**

Sixty five µL of serum was delipidated using Lipisorb and then depleted using a Hu-14, 4.6 x 50mm, Multiple Affinity Removal Column (Agilent Technologies) on an 1100/1200 Agilent LC system. Low abundant proteins were collected in 100% Agilent Buffer A at 0.125mL/min from 4.9-7.3 min and high abundant proteins were eluted to waste with 100% Agilent Buffer B at 1mL/min from 11.51-16 min. Delipidated and depleted serum protein concentration was then determined using a Coomassie Bradford Protein Assay Kit (Thermo Pierce). Proteins were reduced with 10mM Tris(2-carboxyethyl) Phosphine (TCEP) for 30min at 55°C and alkylated with 18.75mM iodoacetamide for 30 min at room temperature in the dark. Proteins were then precipitated overnight in acetone and pellets were reconstituted in 200mM Tetraethylammonium bromide (TEAB) at 1mg/mL and digested with trypsin at 1:40 (trypsin:protein) overnight at 37°C. Tryptic digests were then labeled with Tandem Mass Tag (TMT)10-plex isobaric label reagent set (Thermo Pierce) at 1:1 (peptide:label) ratio for 1hr at room temperature and quenched with 5% hydroxylamine for 15 min before being combined into each respective multi-plex (MP) and dried in a vacuum centrifuge. TMT-labeled MPs were then desalted using C-18 spin columns (Thermo Pierce), dried in a vacuum centrifuge, and stored at -20°C until LCMS analysis.

LC-MS/MS analysis was performed using a Waters nanoAcquity 2D LC system coupled to a Thermo Q Exactive Plus MS. TMT-labeled MPs were resolved over 12 fractions, 90 minute gradient per fraction, and fractionated using two-dimensional reversed-phase chromatography prior to MS analysis. The column eluate was directly introduced into the mass spectrometer via a nano-ESI source and candidate ions were selected and fragmented using a data-dependent Top-15 acquisition method. Full MS survey scans were collected at a resolution of 35,000, scan range of 400-1800 Thompsons (Th; Th = Da/z). MS/MS scans were collected at a resolution of 35,000 with a 1.2 Th isolation window. In order for an ion to be considered a candidate for fragmentation it had to be assigned a charge in the range of +2 to +4.

Raw LC-MS/MS data was then processed using Proteome Discoverer v1.4 (Thermo) by searching a decoy human SwissProt database using the following parameters for both MASCOT and Sequest search algorithms: tryptic peptides with at least 6 amino acids in length and up to two missed cleavage sites, precursor mass tolerance of 10ppm, fragment mass tolerance of 0.02 Da, instrument type: ESI-FTICR, static modifications: cysteine carbamidomethylation, N-terminal TMT-10 plex, and dynamic modifications: asparagine and glutamine deamindation, and methionine oxidation, and lysine TMT-10plex.

**ROC Curve Analysis**

Performance of each of the biomarkers was evaluated by AUC (area under curve) statistics generated from ROC (Receiver Operating Characteristic) curve analysis. Analysis and visualization were done in R using the ROCR libraries (2). Sensitivity was calculated as (Sensitivity = A/(A+C)); Specificity was calculated as Specificity = D/(B+D); Positive predictive value was calculated as PPV = (sensitivity * (1-prevalence))/(((1-sensitivity)*prevalence) + ((1-specificity)*(1-prevalence))); Negative predictive values as calculated as NPV = (specificity * (1-prevalence))/(((1-sensitivity)*prevalence) + ((specificity)*(1-prevalence))) where predicted/reference event = A, predicted event and reference no event = B, predicted no event/reference event = C, predicted no event/reference no event = D. Odds ratios (OR) are reported for logistic regression analyses, with corresponding 95% confidence intervals (CI) and p-values (summary alpha error =0.05, two-sided testing). The threshold of p <0.05 was used to define statistical significance. All statistical analyses were conducted using SAS version 9.4 (Cary, North Carolina).

**References**

1. Lynes MD, Leiria LO, Lundh M, et al. The cold-induced lipokine 12,13-diHOME promotes fatty acid transport into brown adipose tissue. *Nat Med* 2017;23(5):631-637.

2. Kuhn M. Building Predictive Models in R Using the caret Package. *Journal of Statistical Software* 2008;28(5):1-26.

**Supplementary Table S1. Clinico-pathological variable distribution between training and testing cohorts**

| **Variable** | **Training** | **Testing** | **P value** |
| --- | --- | --- | --- |
| **N** | **267** | **115** |  |
| Age at RP (years) |  |  |  |
| Mean (SD) | 58.3 (8.3) | 57.8 (8.5) | 0.547 |
| Race – N (%) |  |  |  |
| CA & Other | 217 (81.3) | 97 (84.4) |  |
| AA | 50 (18.7) | 18 (15.6) | 0.471 |
| Pathological T stage- N (%) |  |  |  |
| pT2 | 183 (72.6) | 76 (73.1) |  |
| pT3-4 | 69 (27.4) | 28 (26.9) | 0.929 |
| Gleason sum- N (%) |  |  |  |
| 3+3 | 130 (52.4) | 66 (58.4) |  |
| 3+4 | 73 (29.4) | 32 (28.3) |  |
| 4+3/8-10 | 45 (18.2) | 15 (13.3) | 0.204 |
| Surgical margin -N (%) |  |  |  |
| Negative | 223 (84.2) | 88 (76.5) |  |
| Positive | 42 (15.8) | 27 (23.5) | 0.076 |
| BCR -N (%) |  |  |  |
| No | 217 (81.3) | 93 (80.9) |  |
| Yes | 50 (18.7) | 22 (19.1) | 0.926 |
| FU (years) |  |  |  |
| Median (range) | 7.0 (0.2-18.6) | 6.6 (0.2-16.4) | 0.333 |

**Supplementary Fig. S1.**

**Kaplan-Meier BCR-free surival curves across dichotomized marker groups**


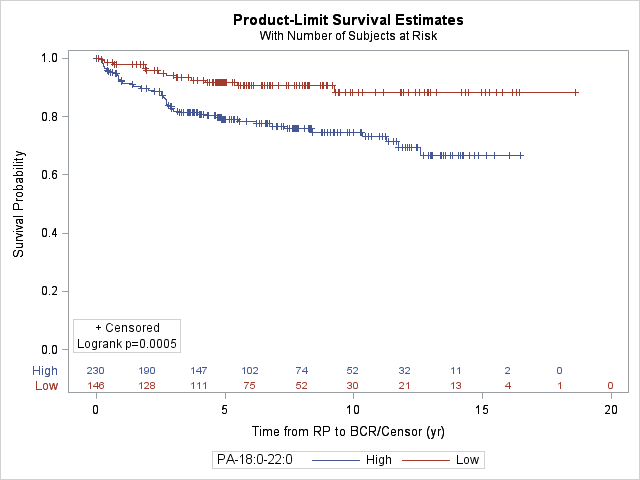

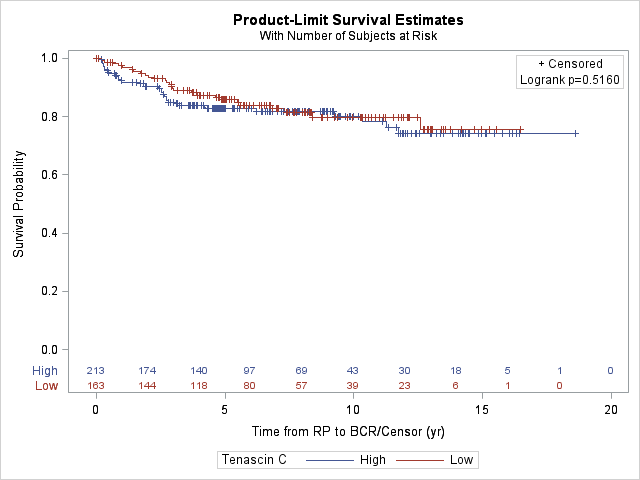

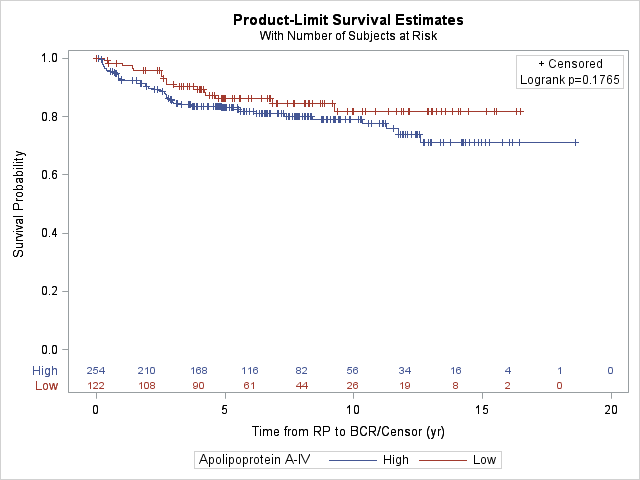

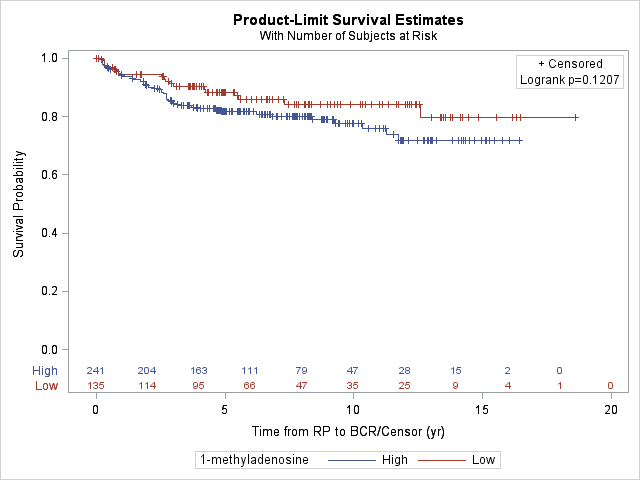


**Supplementary Fig. S2.**

**Kaplan-Meier metastasis-free surival curves across dichotomized marker groups**
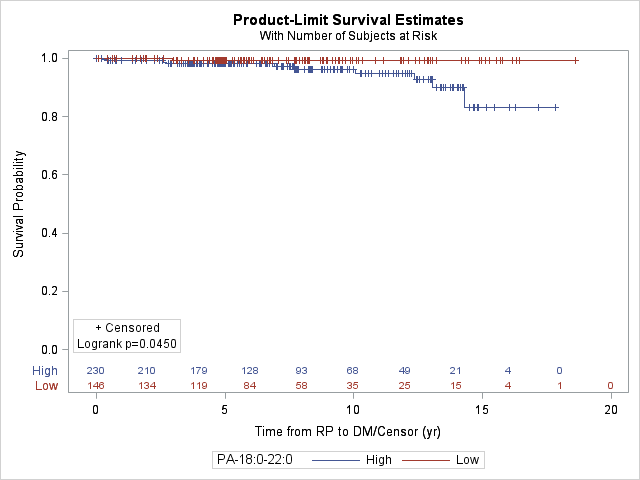

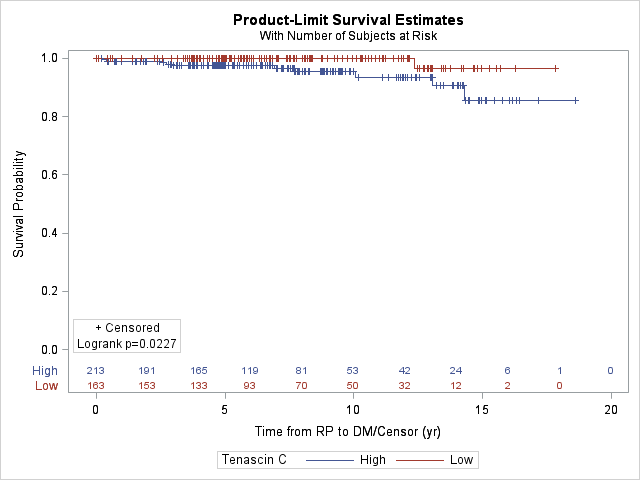


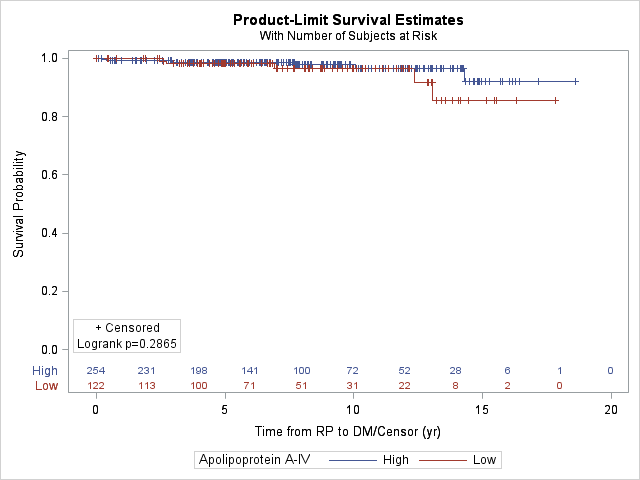

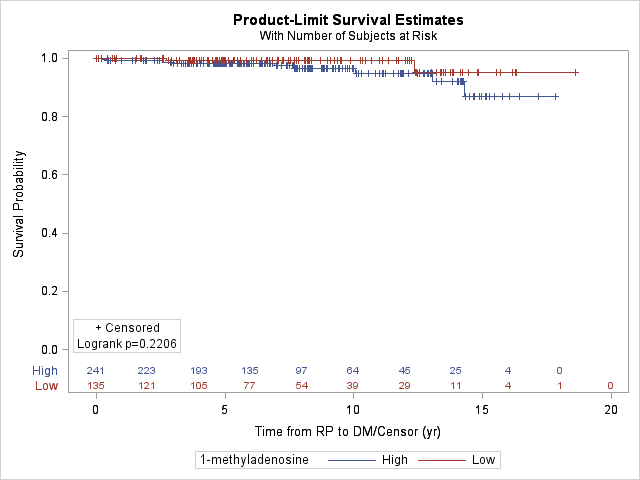

Supplement: Supplementary file 1 — Additional file 1: Figure S1. Kaplan–Meier BCR-free survival curves across dichotomized marker levels of (PA)18:0-22:0, Tenascin C, Apolipoprotein A-IV and 1-Methyladenosine. Figure S2. Kaplan–Meier metastasis-free survival curves across dichotomized marker levels of (PA)18:0-22:0, Tenascin C, Apolipoprotein A-IV and 1-Methyladenosine. Table S1. Clinico-pathological variable distribution between training and testing cohorts. [file 12967_2019_2185_MOESM1_ESM.docx]
